# Supplementary material for: Temporally-precise disruption of prefrontal cortex informed by the timing of beta bursts impairs human action-stopping
Source: Neuroimage. Author manuscript; Available in PMC 2020 Dec 15. (PMC7736218; doi:10.1016/j.neuroimage.2020.117222)
Supplement: S1 Table [file NIHMS1639041-supplement-S1_Table.pdf]

Supplementary Table 1. Peak beta frequency in the $Stop_{Win}$ for each participant

Participant	Peak Beta (Hz)
S1	13.75
S2	14.50
S3	13.50
S4	13.00
S5	13.00
S6	13.00
S7	13.25
S8	21.00
S9	13.00
S10	15.50
S11	13.00
S12	13.00
S13	13.00
