# Supplementary material for: Temporally-precise disruption of prefrontal cortex informed by the timing of beta bursts impairs human action-stopping
Source: Neuroimage. Author manuscript; Available in PMC 2020 Dec 15. (PMC7736218; doi:10.1016/j.neuroimage.2020.117222)
Supplement: S1 Fig [file NIHMS1639041-supplement-S1_Fig.pdf]

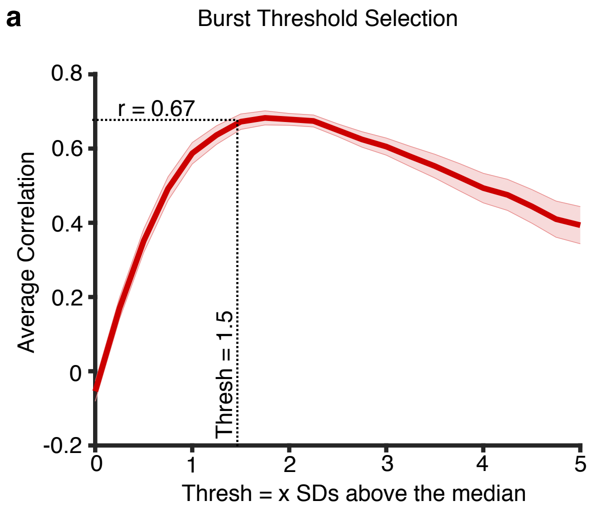

Supplementary figure 1: Relationship between beta amplitude and burst count across different threshold criteria. A high correlation is seen for our selected threshold (median + 1.5*S.D.).
