# Supplementary material for: Temporally-precise disruption of prefrontal cortex informed by the timing of beta bursts impairs human action-stopping
Source: Neuroimage. Author manuscript; Available in PMC 2020 Dec 15. (PMC7736218; doi:10.1016/j.neuroimage.2020.117222)
Supplement: S2 Fig [file NIHMS1639041-supplement-S2_Fig.pdf]

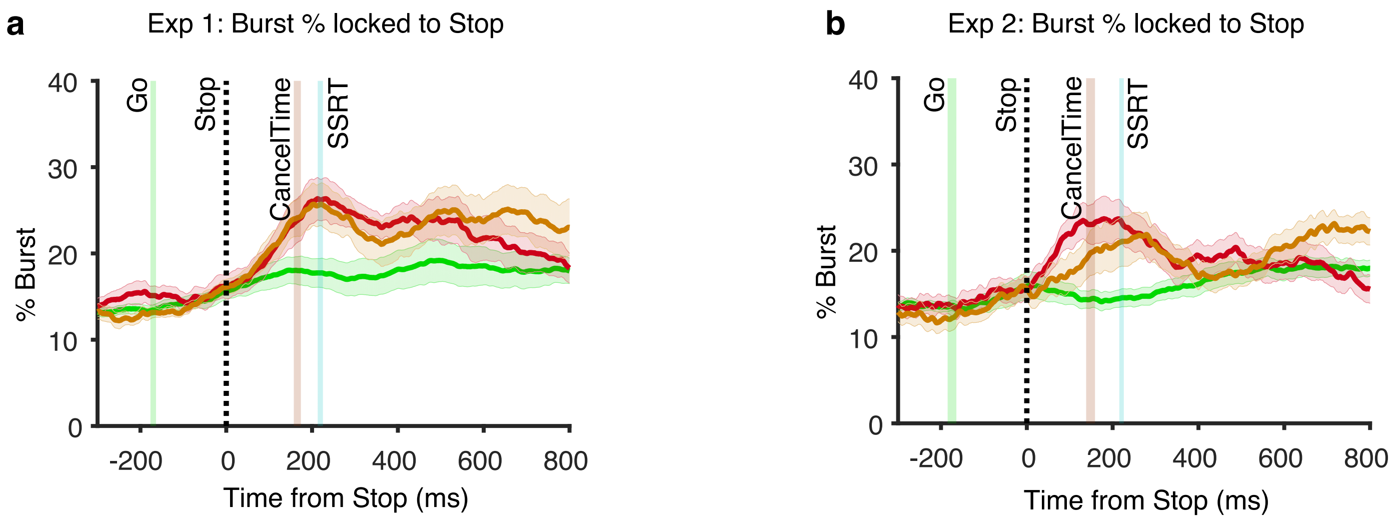

Supplementary figure 2: Burst % across time for all trial types. A) Burst % for Successful (red), Failed (orange) and Correct Go (green) trials for Experiment 1. Both successful and failed stop trials show increase in burst % prior to SSRT. B) Same but for Experiment 2.
