# Supplementary material for: Temporally-precise disruption of prefrontal cortex informed by the timing of beta bursts impairs human action-stopping
Source: Neuroimage. Author manuscript; Available in PMC 2020 Dec 15. (PMC7736218; doi:10.1016/j.neuroimage.2020.117222)
Supplement: S3 Fig [file NIHMS1639041-supplement-S3_Fig.pdf]

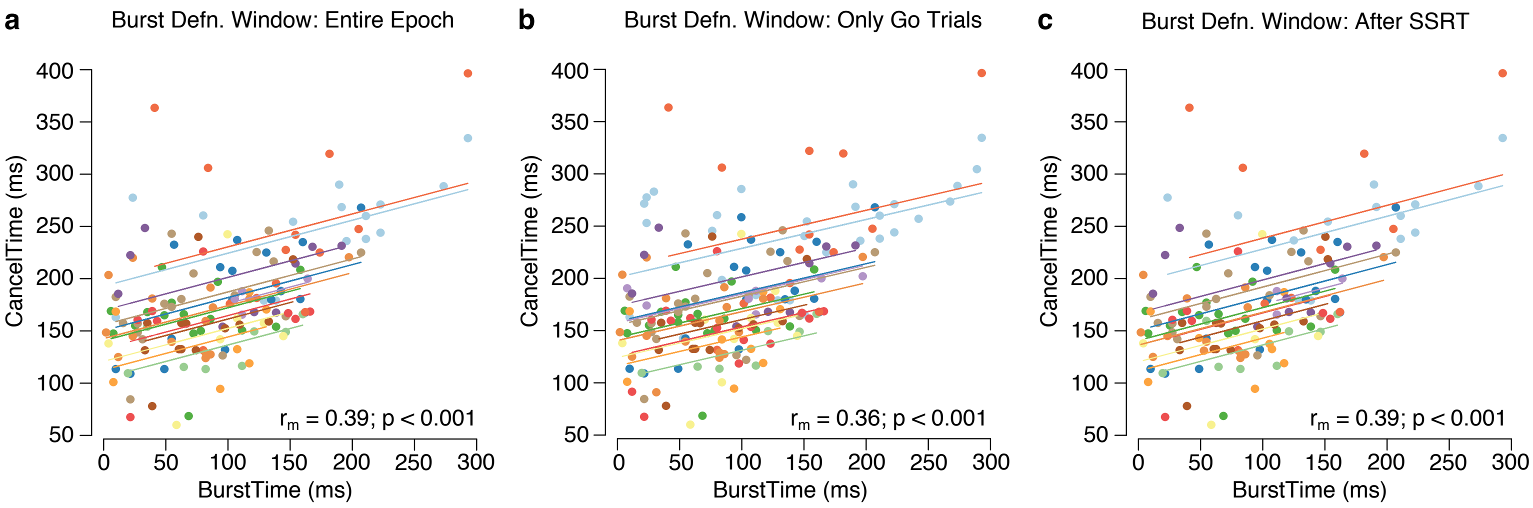

Supplementary figure 3: Single trial relationship between BurstTime and CancelTime for different burst definition windows. a) Using the entire epoch, b) using the period prior to the Go (-1000 to -500 ms in relation to the stop signal) only for the Go trials, and c) using a window after SSRT (500 to 1000 ms in relation to the stop signal).
