# Supplementary material for: Temporally-precise disruption of prefrontal cortex informed by the timing of beta bursts impairs human action-stopping
Source: Neuroimage. Author manuscript; Available in PMC 2020 Dec 15. (PMC7736218; doi:10.1016/j.neuroimage.2020.117222)
Supplement: S4 Fig [file NIHMS1639041-supplement-S4_Fig.pdf]

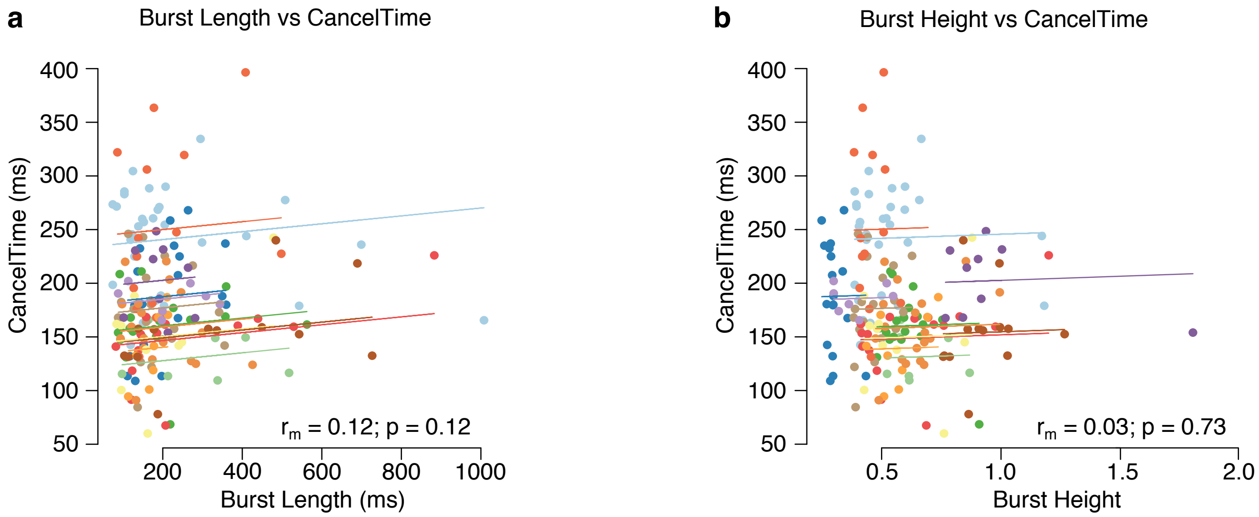

Supplementary figure 4: Relationship between other burst parameters and CancelTime. a) Repeated measures correlation between burst duration/length and CancelTime. b) Same for burst height and CancelTime. There is no reliable relationship between these burst parameters and CancelTime.
